# Formation of Nanolaminated Structure with Enhanced Thermal Stability in Copper

**DOI:** 10.3390/nano11092252

**Published:** 2021-08-31

**Authors:** Jianxin Hou, Xiuyan Li, Ke Lu

**Affiliations:** Shenyang National Laboratory for Materials Science, Institute of Metal Research, Chinese Academy of Sciences, Shenyang 110016, China; jxhou15s@imr.ac.cn (J.H.); lu@imr.ac.cn (K.L.)

**Keywords:** nanolaminated structure, deformation mechanism, low angle grain boundaries, thermal stability, copper

## Abstract

Nanolaminated structure with an average boundary spacing of 67 nm has been fabricated in copper by high-rate shear deformation at ambient temperature. The nanolaminated structure with an increased fraction of low angle grain boundaries exhibits a high microhardness of 2.1 GPa. The structure coarsening temperature is 180 K higher than that of its equiaxial nanograined counterpart. Formation of nanolaminated structure provides an alternative way to relax grain boundaries and to stabilize nanostructured metals with medium to low stacking faults energies besides activation of partial dislocations.

## 1. Introduction

Formation of laminated structure in nanoscale (boundary spacing < 100 nm) with low angle grain boundaries (GBs) provides an effective way to stabilize nanostructured metals and to achieve extraordinary grain refinement. By using surface mechanical grinding treatment (SMGT) with high strain rates and high strain gradients, nanolaminated (NL) structure in Ni can be refined to a length scale as small as 20 nm [1], which is one order magnitude below the saturated grain size induced by traditional plastic deformations [2,3,4]. The 20-nm lamellae in Ni exhibit superior thermal stability, with its onset temperature for grain coarsening (T_on_) 40 K higher than that of saturated ultrafine-grained structure. Similarly, extraordinary grain refinement and/or enhanced thermal stability has been achieved through the formation of NL structure in other metals or alloys with high stacking faults energies (SFE) such as pure Al [5], interstitial-free steel [6,7], Al-Cu [8,9] and Al alloy 5052 [10]. Even though the detailed mechanism remains ambiguous, formation of NL structure can be attributed to extensive dislocation activities during plastic deformation with high strain rates and strain gradients [5,9,11,12], where large amount of geometrically necessary dislocations (GNDs) would be generated and line up as laminated boundaries to accommodate increasing number of dislocations [4,13], finally forming laminated structure in nanoscale.

However, for those metals or alloys with medium to low SFE, pure Cu for example, NL structure can hardly be detected except for the localized regions in the shear bands [14]. Experimental observations show that laminated structure can be induced in Cu by severe plastic deformation (SPD), such as accumulative roll bonding (ARB) [15,16,17] and equal channel angular pressing (ECAP) [17,18]. Nevertheless, the boundary spacing between lamellae is usually as large as 200–300 nm, which is corresponding to the critical grain size for dislocation storage [19]. Further refining lamellae below the saturation size is challenging due to the increasing tendency of GB annihilation via dislocation annihilation and migration of GBs during straining [20]. By increasing strain rates and/or decreasing deformation temperature, grains of pure Cu can be refined to 40 nm [21,22,23] and even down to 10 nm [24]. However, the nanostructured Cu tends to show morphology of roughly equiaxed grains with plenty of twins and stacking faults rather than laminated structure as that in Ni [1,11] and Al [5]. Detailed analysis shows that below 70 nm, full dislocation activities in Cu may be inhibited while partial dislocation activities become more favorable [21,22]. As a result, NL structure which can only be fulfilled with full dislocation behaviors is impeded. To obtain NL structure in Cu need to hinder partial dislocation activities while enhancing the laminated boundary generation by inducing higher density of GNDs.

In addition to grain size, deformation conditions, such as temperature and strain rate, et al., also affect deformation mechanism significantly [23], which provides a possible way to generate NL structure in Cu with boundary spacing smaller than 200 nm. In our recent study about tension induced GBM in nanograined (NG) Cu [25], it has been found that full dislocation motion plays a key role in mechanically induced GBM and results in an increasing fraction of low angle GBs. In other word, although the deformation in Cu with grain size range from ~200 nm to 70 nm can be dominated by mechanically induced GBM, low angle GBs are still generating through full dislocation motions in this process. In this study, SMGT was employed to try to generate NL structure in Cu. The process was conducted at room temperature (RT) rather than liquid nitrogen temperature (LNT) to pump-in full dislocations as many as possible. High strain rates of SMGT facilitates dislocation generation, while a modest temperature is beneficial for suppressing partial dislocation activities and full dislocation annihilation as well. Additionally, high density of GNDs can be induced by high strain gradient during SMGT [26]. As NL structure is fabricated in Cu, its stability has also been identified.

## 2. Materials and Methods

Fully recrystallized coarse-grained oxygen-free pure Cu (99.97 wt.%) rods with an average grain size of 25 μm were used in this work. The rods were machined to a diameter of ~10 mm and then subjected to SMGT (BYJC-OKUMA, Beijing, China) for 7 passes. The principle and setup of the SMGT were introduced by previous works [5,21,22,25]. During SMGT processing, the rod sample rotated at a velocity of 100 r/min while a polished hemispherical WC/Co tip (Φ = 6 mm) penetrated the sample surface by 40 μm deep and slid along the sample at a velocity of 2.5 mm/min. The sample was cooled by water rather than liquid nitrogen to maintain ambient temperature. The roughly equiaxed NG sample in Ref. [25] processed by SMGT at liquid nitrogen temperature (LNT) is also included for comparison.

The treated surfaces of the as-prepared samples were protected by electro-plating a pure Cu film coating before microhardness test and annealing process. The samples were then annealed at different preset temperature for 30 min and air-cooled down to room temperature for thermal stability investigation. Samples were mechanical grinded and then electrolytic polished before cross-sectional microstructure observations and in-depth microhardness measurements.

Microhardness of the treated sample was measured on a Qness Q10 A+ Automatic Vickers Hardness Tester (QATM, Golling, Austria) with a load of 20 g and a loading duration of 10 s. In-depth microhardness distributions were measured from the cross-sectional view, i.e., on the surface vertical to the transversal direction of the rod. Microhardness of the topmost surface layer was measured from the planar view, i.e., on the surfaces vertical to the normal direction of the rod.

Cross-sectional morphology observation of the as-prepared and the as-annealed samples were carried out on an FEI Verios 460 scanning electron microscope (SEM) (FEI Inc., Hillsboro, OR, USA) operated at 18 kV. Orientation information was determined by using electron backscatter diffraction (EBSD) equipped on an FEI Nova NanoSEM 450 (FEI Inc., Hillsboro, OR, USA) with a step size of 20 nm. Detailed microstructure was characterized by an FEI Talos F200X field emission gun transmission electron microscope (TEM) (FEI Inc., Hillsboro, OR, USA) operated at 200 kV. TEM foils were mechanically thinned down to 40 μm, followed by final thinning by using double-jet electrolytic polishing.

## 3. Results

**Figure 1:**
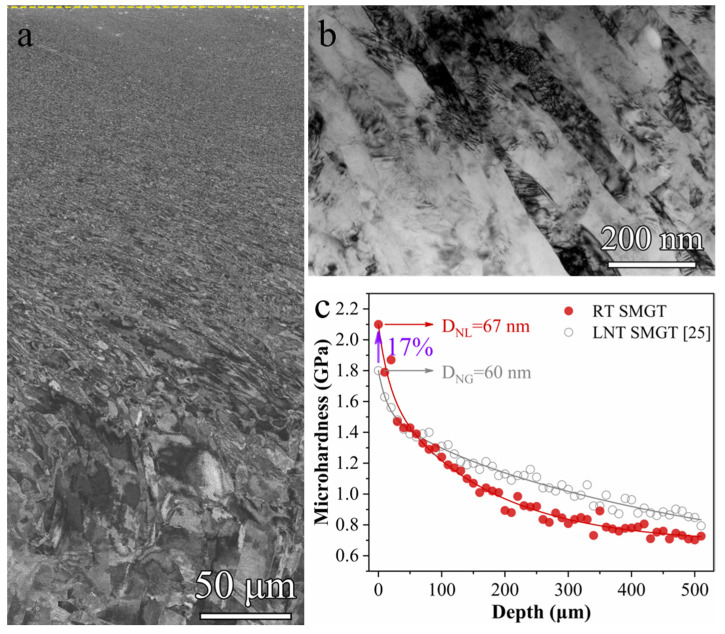
(**a**) Typical SEM image showing the gradient microstructure of SMGT processed Cu. (**b**) TEM image of the NL structure at a depth of ~5 μm from the treated surface. (**c**) Microhardness distribution of the sample processed by SMGT at RT and at LNT [25].

Typical gradient microstructure is induced in the surface layer of coarse-grained Cu after SMGT processing (as shown in Figure 1a) owing to the gradient distribution of plastic strain and strain rates [1,9]. It should be noted that the topmost 1-μm-thick layer was removed from our characterization to avoid any potential contamination and surface effects. In the top 10 μm-thick surface layer, NL structure is formed with lamellae thickness mainly ranging from 30 nm to 120 nm (Figure 1b), averagely 67 ± 25 nm, which is different from the NG sample processed by SMGT at cryogenic temperature where nano-sized grains are almost equiaxed [22,25]. Deformation twins can rarely be observed within the nano-laminates. The laminated boundaries are up to several microns in length, giving an estimated grain aspect ratio of about 10–20, such as those observed in the surface layer of other metals and alloys, such as Ni, Fe, Al and Al alloy [1,5,7,9]. Corresponding to the microstructural gradients, a gradient distribution of microhardness can be detected in the deformed layer, as plotted in Figure 1c. The NL layer exhibits a high hardness of 2.1 GPa, 17% higher than that of NG sample with similar grain size [25].

**Figure 2:**
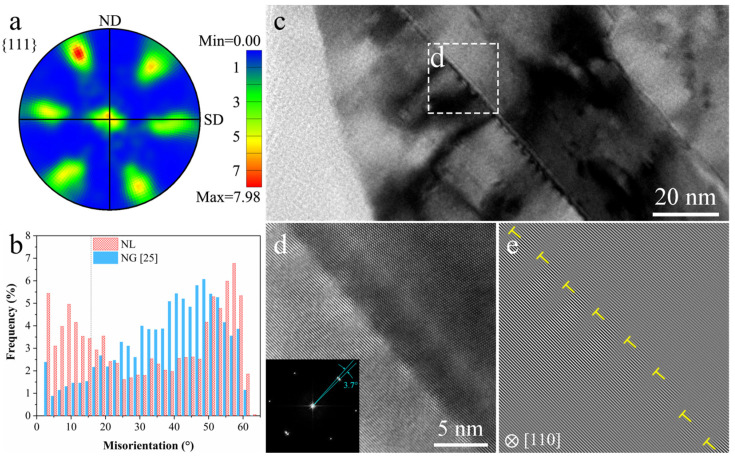
(**a**) {111} pole figure obtained from EBSD data of the NL sample. (**b**) Misorientation distribution of the NL and NG sample [25]. (**c**) Typical bright field TEM image showing the faceting boundary between two lamellae. (**d**) High-resolution TEM image of white rectangular area in (**c**) and its corresponding FFT pattern. (**e**) Corresponding one-dimensional {110} fringes of (**d**), showing the presence of regularly arranged full dislocations.

The {111} pole fifig (Figure 2a) shows that the texture components of NL structure are major {111}<112>, suggesting a strong simple shear deformation in the top surface layer during processing. Misorientation distributions of NL sample and NG sample (Figure 2b) show that the fraction of low angle GBs in the NL sample (~30%) is significantly higher than that of NG sample (~8.5%). A typical bright field TEM image illustrated in Figure 2c delineates the straight and sharp boundary dividing two lamellae. The boundary boxed by a white rectangular is further magnified in Figure 2d, which shows the atomic lattice structures of the two lamellae across the boundary. The boundary is faceted at atomic level, similar as the GB structure observed in Refs. [1,5,11], which is resulted from adjustment of boundary structures for lowering excess energy. A minor orientation change of 3.7° can be measured from the inserted fast Fourier transformation (FFT). One-dimensional {110} fringes (slip planes) are shown in Figure 2e, revealing the presence of the regularly arranged dislocations. It can be counted that a full dislocation appears every 15–16 atomic plane, suggesting a misorientation of 3.6–3.8° (arctan 1/16–arctan 1/15) according to geometric model, which is consistent with that measured by FFT and high-resolution TEM.

By properly adjusting the deformation parameters (decreasing strain rates and increasing deformation temperature) during the SMGT process, full dislocation motions (GBM) are significantly facilitated although little deformation twinning occurs (corresponding to a twin boundary fraction of 4.6%, counted by EBSD). The thickness of grains with sizes ranging from ~200 nm to 70 nm (where pronounced mechanically induced GBM happens) also increases from 50 μm in the LNT-SMGT sample [21] to more than 100 μm in the RT-SMGT sample, suggesting that mechanically induced GBM, which is related to full dislocation motion [25], plays a more important role in the RT deformation process. The pronounced full dislocation activities are further evidenced by the substantial texturing among the NL structure (Figure 2a). Even though mechanically induced GBM generally causes grain coarsening and hinders the processing of nanograined materials by plastic deformation [21], laminated structure is refined to nanoscale as high density of GNDs is generated and accumulated in the topmost surface layer with high strain gradients (0.63 μm^−1^) and high strain rates (10^2^–10^3^ s^−1^). Formation of NL structure can be attributed to the increased fraction of low angle GBs generated by mechanically induced GBM [25].

**Figure 3:**
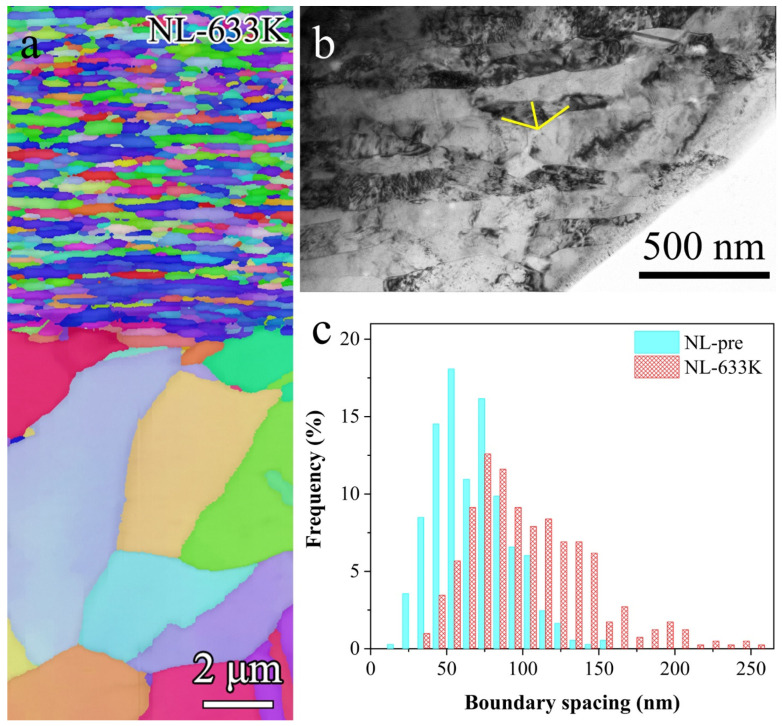
(**a**) EBSD image of the top 20 μm surface layer after annealing at 633 K. (**b**) Typical bright field TEM image of the NL sample annealed at 633 K. (**c**) Boundary spacing distribution of the as-prepared NL sample and the as-annealed (633 K) one.

Thermal stability of the NL specimen was examined by annealing at different temperatures for 30 min. No obvious change is observed in either the morphology or the boundary spacing of NL after annealing at 593 K. As annealing temperature reaches 633 K, although no apparent recrystallization can be detected from the EBSD image (Figure 3a), shortening and fragmentation of the nanolaminates can be detected (as shown in Figure 3b), which is frequently observed in the structural coarsening process of NL structure [27]. Statistical measurements of TEM (Figure 3c) show that average thickness of the NL layer increases slightly from 67 ± 25 nm in the as-prepared samples to 104 ± 40 nm in the as-annealed ones (633 K). By contrast, the original ultrafine structures in deeper layers beneath the NL structure coarsened into micrometer-sized grains under the same annealing condition (Figure 3a).

**Figure 4:**
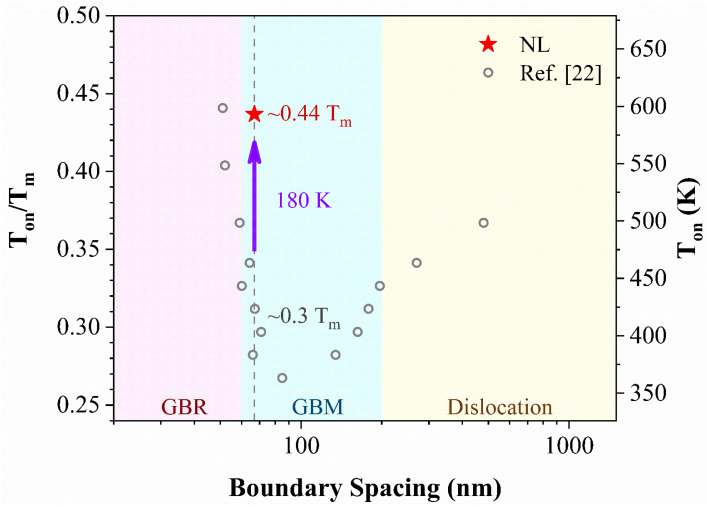
Measured onset temperature for grain coarsening (T_on_) as a function of boundary spacing.

Previous studies [21,22,28,29] have shown that stability of pure metals and degree of GBM are grain size dependent. With the decrease of grain size, stability of the grains generally decreases and GBM process becomes more pronounced due to the increasing density of GBs with high mobility. However, when the grain size is below ~70 nm, autonomously GB relaxation (GBR) process accompanied with transition of deformation mechanism is triggered by the interaction of GBs and partial dislocations [22]. As a result, stability of pure Cu increases with the decreasing grain size, as plotted in Figure 4. The 10-nm Schwarz crystal in Cu is even stable against grain coarsening when close to the equilibrium melting point [24]. Although intensive boundary relaxation can be triggered by rapid heating [30], deformed grains of about 70 nm in size show the worst stability and grain coarsening occurs when annealed at temperature lower than 0.3 T_m_. However, the present NL structure with thickness equivalent to the most unstable grain size exhibits excellent thermal stability, with its T_on_ is 180 K higher than that of its equiaxed NG counterpart (Figure 4). Moreover, the NL structure also exhibits an enhanced mechanically structural stability against grain coarsening, which can be reflected by the elevated microhardness of 2.1 GPa. The value of microhardness is even higher than that of Hall-Petch relationship extrapolated from the coarse grained Cu [31], which is understandable due to the presence of defects within the laminates (Figure 1b). The nanostructured pure Cu with notably instable sizes is stabilized by pumping in more GNDs and forming low energy laminated boundaries. It is conceivable that intensive GBR, originating from the generation of nanolamellae with atomically ordered boundaries, is triggered by full dislocation slips during SMGT deformation at ambient temperature. Formation of NL structure with increased fraction of low angle boundaries provides an alternative way to relax GBs and to stabilize nanostructured Cu besides activation of partial dislocations in plastic deformation.

## 4. Conclusions

NL structure with an average boundary spacing of 67 nm is fabricated in Cu with medium to low SFE through SMGT process at ambient temperature. Formation of textured NL structure with an increased fraction of low angle boundaries can be attributed to the full dislocation dominated deformation with high shear strain rates and strain gradients. The NL Cu with elevated microhardness of 2.1 GPa exhibits an enhanced thermal stability, with its T_on_ is 180 K higher than that of the equiaxed NG counterpart. Formation of NL structure provides an alternative way to stabilize nanostructured metals with medium to low SFE.

## Data Availability

Data available on request due to restrictions, e.g., privacy or ethics.
